# Fatty acid-balanced oil improved nutrient digestibility, altered milk composition in lactating sows and fecal microbial composition in piglets

**DOI:** 10.5713/ab.23.0359

**Published:** 2024-02-28

**Authors:** Yu Zhang, Shuyu Peng, Shuang Dong, Jihua Wang, Yu Cao, Yongxi Ma, Chunlin Wang

**Affiliations:** 1 State Key Laboratory of Animal Nutrition, College of Animal Science and Technology, China Agricultural University, Beijing 100193, China; 2 CALID BIOTECH (WUHAN) CO., LTD, Wuhan 430073, China

**Keywords:** Fatty Acid, Lactating Sows, Microbiota, Milk Composition, Nutrient Digestibility, Serum Indexes

## Abstract

**Objective:**

This study aimed to investigate the effects of dietary supplementation of a fatty acid-balanced oil, instead of soybean oil, on reproductive performance, nutrient digestibility, blood indexes, milk composition in lactating sows, and fecal microbial composition in piglets.

**Methods:**

Twenty-four sows (Landrace×Yorkshire, mean parity 4.96) were randomly allotted to two treatments with twelve pens per treatment and one sow per pen based on their backfat thickness and parity. The experiment began on day 107 of gestation and continued until weaning on day 21 of lactation, lasting for 28 days. The control group (CG) was fed a basal diet supplemented with 2% soybean oil and the experimental group (EG) was fed the basal diet supplemented with 2% fatty acid-balanced oil.

**Results:**

The fatty acid-balanced oil supplementation increased (p<0.05) the apparent total tract digestibility of dry matter, crude protein, and gross energy in sows. The lower (p<0.05) serum high-density lipoprotein cholesterol and albumin levels of sows were observed in the EG on day 21 of lactation. Dietary supplementation with the fatty acid-balanced oil decreased the fat content, increased the immunoglobulin G level, and changed (p<0.05) some fatty acid content in milk. Moreover, the fatty acid-balanced oil supplementation changed (p<0.05) the fecal microbial composition of piglets, where the average relative abundance of *Spirochaetota* was decreased (p<0.05) by 0.55% at the phylum level, and the average relative abundance of some potentially pathogenic fecal microorganism was decreased (p<0.05) at the species level.

**Conclusion:**

The fatty acid-balanced oil improved nutrient digestibility, changed the serum biochemical indices and milk composition of sows, and ameliorated the fecal microbial composition of piglets.

## INTRODUCTION

Sows often do not get enough energy from feed and need to mobilize body reserves to maintain milk production to support litter growth [1]. Dietary oils help achieve higher energy density in diets for lactating sows to increase the total energy intake, better meets the energy needs for milk production to raise their progeny, while minimizing catabolic depletion of the sow’s body reserves [2,3]. A study has shown that 3.8% to 3.9% oil supplementation in sows’ diet could improve the reproductive performance of sows and increase the levels of milk fat and milk immunoglobulin [4].

The fatty acid composition of different oil sources is quite different, and the use of a single source of oil usually cannot meet the needs of animals for fatty acids. For example, the fatty acid composition of soybean oil is relatively simple, mainly n-6 polyunsaturated fatty acid (PUFA), lack of n-3 PUFA, medium chain fatty acid (MCFA) and other functional fatty acids [5]. n-3 PUFA is related to the quality of ovarian follicles, embryo survival and subsequent litter size [6]. Medium chain fatty acids (MCFA), considered as antibiotic replacers, have strong antibacterial activity against Gram-positive cocci and *Escherichia coli* [7]. Supplementation of n-3 PUFA and MCFA has been shown to be beneficial to sows, so soybean oil may lack some fatty acids that sows need. The balance of fatty acids mainly includes the balance of essential and non-essential fatty acids, the balance of saturated and unsaturated fatty acids, the balance of n-3 and n-6 PUFA, and the balance of short chain fatty acids and MCFA [8–10]. Yao et al [11] reported the suitable n-6:n-3 PUFA ratio in sow diet can improve daily gain and immune performance of suckling piglets. Chen et al [12] reported during late pregnancy and lactation, providing a sow diet containing MCFA shortened the weaning-to-estrus interval; dietary n-3 PUFA supplementation increased the fat and protein content in the colostrum; and providing a sow diet with sodium butyrate promoted intestinal health and decreased the pre-weaning mortality of suckling piglets; dietary sodium butyrate, MCFA, or n-3 PUFA supplementation favored improvements in the intestinal microbiota.

However, no studies have systematically investigated the ideal fatty acid pattern for lactating sows. Because the milk and body fatty acid composition of sows are different with those in soybean oil, our hypothesis is that using fatty acid-balanced oil instead of soybean oil, may affect reproductive performance, nutrient digestibility, blood indexes, and milk composition of lactating sows, and beneficial to growth performance of piglets.

## MATERIALS AND METHODS

The experimental design and procedures used in this study were approved by the animal ethical committee of China Agricultural University (Beijing, China; No. AW90602202-1-2) according to the Chinese Guidelines for Animal Welfare. The experiment was conducted at the FengNing Swine Research Unit of China Agricultural University (Chengdejiuyun Agricultural and Livestock Co., Ltd., Hebei, China).

### Animals and experimental design

A total of 24 sows (Landrace×Yorkshire, mean parity 4.96) were randomly allotted into two treatments with 12 replicates (1 sow per replicate pen) per treatment based on their backfat thickness and parity. The two treatments included a corn-soybean meal-based diet supplemented with 2% soybean oil (control group, CG) and the corn-soybean meal-based diet supplemented with 2% fatty acid-balanced oil (experimental group, EG). The fatty acid-balanced oil used in this research was provided by CALID BIOTECH (WUHAN) CO., Ltd. It is the product of mixing various oils and fats. The composition of soybean oil and the fatty acid-balanced oil used in this research is shown in Table 1. Compared with soybean oil, the fatty acid-balanced oil was supplemented with C4:0, C12:0, C13:0, C20:3n6, C20:4n6, C20:3n3, C20:5n3, C24:1, and C22:6n3, and adjusted the proportion of other fatty acids.

The experiment began on day 107 of gestation and continued until weaning on day 21 of lactation, lasting for 28 days. The diets (Table 2) were formulated to meet the recommended requirements of lactating sows [13].

### Feeding and management

Every sow was transferred to an individual experimental pen in the delivery room after bathing and disinfection on day 107 of gestation. Dietary treatments were started on day 107 of gestation and sows were fed by dry feed mixed with water three times a day at 0530, 1030, and 1630 h. The sows were fed 2.76 kg a day before delivery, with no feeding on the day of delivery. No refusal to feed was observed before delivery. After delivery, gradually increased daily feeding until *ad libitum* intake on an individual sow basis. All sows were kept in experimental pens with relatively constant humidity of 65%. The temperature in the delivery room was controlled at 20°C to 23°C. The sows had an *ad libitum* access to water during the experiment period.

### Reproductive performance

On the day of delivery and day 21 of lactation, all piglets were weighed individually, and residual feed was collected and weighed in each pen. Backfat thickness at the P2 site was measured using the ultrasonic backfat meter (Piglog105; SFK Technology A/S, Herlev, Denmark) on the day of delivery and day 21 of lactation to calculate the backfat loss of lactating sows. Average daily feed intake (ADFI) of sows, average daily gain (ADG) of piglets, born alive, number weaned, litter birth weight, weaning litter size, litter weight gain, piglet birth weight, and piglet weaning weight were recorded. The reproductive performance of the subsequent litter and the wean-to-estrus interval were observed.

### Nutrient digestibility

During this experiment, approximately 2.0 kg representative feed samples were separately taken from each treatment. Approximately 400 g of feces of sows (six samples per treatment) were collected on day 21 of lactation and dried at 65°C for 72 h. The feed and dried fecal samples were ground through a 60-mesh sieve. The contents of dry matter (DM), crude protein (CP), crude fat (CF) and acid-insoluble ash (AIA) in diet and feces were determined according to the Association of Official Analysis Chemist [14]. A PARR-6400 (Calorimeter; PARR Instrument Company, Moline, IL, USA) automatic oxygen bomb calorimeter was used to measure gross energy (GE). The formula for calculating the apparent total tract digestibility (ATTD) of nutrients is as follows:


$$\text{ATTD of nutrients }(\%)=[1-({\text{AIA}}_{\text{diet}}\times {\text{Nutrient}}_{\text{feces}})/({\text{AIA}}_{\text{feces}}\times {\text{Nutrient}}_{\text{diet}})]\times 100$$

The concentrations of AIA in the diets of CG and EG were 0.19% and 0.18%, respectively.

### Serum biochemical indexes

Blood samples of 5 mL (six samples per treatment) were obtained from the sows’ auricular vein on the day of delivery and day 21 of lactation, and blood samples of 5 mL (six samples per treatment) were obtained from the piglets’ anterior vena cava on day 21 of lactation. Within 30 min after samples collection, the serum was obtained by centrifugation at 3,000×*g* for 15 min and then immediately stored at −20°C for further analysis. Serum samples were analyzed for serum biochemical indexes: triglyceride, total cholesterol, high-density lipoprotein cholesterol (HDL-C), low-density lipoprotein cholesterol, albumin (ALB), and total protein; liver functions: aspartate aminotransferase, alanine aminotransferase; antioxidant indexes: glutathione peroxidase (GSH-Px), total antioxidant capacity (T-AOC), superoxide dismutase (SOD), catalase, and malondialdehyde (MDA); immunoglobulins: immunoglobulin A (IgA), IgG and IgM; inflammatory factors: tumor necrosis factor-α (TNF-α), interleukin-8 (IL-8), IL-6, and IL-10. Serum biochemical and liver function indexes were determined by colorimetric method and automatic biochemical analyzer (Hitachi 7020, Tokyo, Japan). The content of MDA and the activity of T-AOC, GSH-Px, and T-SOD were assayed using the thiobarbituric acid reaction, the xanthine oxidase, the dithiodinitrobenzoic acid colorimetric, and ammonium molybdate method, respectively. Serum TNF-α, IL-8, IL-6, and IL-10 concentrations were calculated by establishing a standard curve according to the commercial ELISA kit (Nanjing Jiancheng Bioengineering Institute, Nanjing, China). All indexes were analyzed by kit according to the manufacturer’s instructions (Nanjing Jiancheng Bioengineering Institute, China).

### Milk composition

Within 2 h of starting delivery, 50 mL colostrum (six samples per treatment) was collected from the 2nd, 3rd, and 4th pairs of mammary glands, and 50 mL of milk (six samples per treatment) was collected after 10 IU of oxytocin was injected through the ear vein on day 21 of lactation. After sample collection, all samples were immediately frozen at −20°C. Colostrum and milk were analyzed for milk fat, lactoprotein, and lactose; IgA, IgG, and IgM; fatty acid composition. Milk fat, lactoprotein, and lactose content were tested by infrared spectroscopy (MilkoScan FT2 instrument; Foss MilkoScan, Hillerød, Denmark). Concentrations of IgA, IgG, and IgM levels were determined by immunoturbidimetry according to the steps of the immunoglobulin specific kit (Shandong Sanwei Bioengineering Co., LTD., Qingdao, China) according to methods described [15].

### Fecal microbial composition of piglets

On day 21 of lactation, piglets with a near-average weight were selected to collect about 1 g of fresh feces (six samples per treatment) by rectal palpation and immediately stored in liquid nitrogen for microbial composition analysis. Total microbial genomic DNA extraction was performed according to the E.Z.N.A. soil DNA kit (Omega Bio-tek, Norcross, GA, USA). The hypervariable region V3-V4 of the bacterial 16S rRNA gene was amplified with primer pairs 338F (5′-AC TCCTACGGGAGGCAGCAG-3′) and 806R(5′-GGACTAC HVGGGTWTCTAAT-3′) with the following amplification program (95°C for 3 min, followed by 27 cycles of 95°C for 30 s, 55°C for 30 s, and 72°C for 45 s, and final extension at 72°C for 10 min). Sequencing was performed using Illumina’s Miseq PE300 platform (Illumina, San Diego, CA, USA). Raw tags were obtained by merging paired-end reads using the FLASH (, version 1.2.11/) software. Then the optimized sequences were clustered into operational taxonomic units (OTUs) using UPARSE (, version 7.1) with 97% sequence similarity level. After being rarefied, taxonomic annotation was analyzed using RDP classifier () and the taxonomic composition of the bacterial community was then analyzed.

### Statistical analysis

Each litter was considered the experimental unit to analyze born alive, number weaned, litter birth weight, litter weight gain, piglets birth weight, and piglets weaning weight. Individual sows or piglets were treated as the experimental unit to analyze the other indexes. The normality of residuals and equal variances was checked using the UNIVARIATE procedure of SAS 9.4 (SAS Institute, Cary, NC, USA). Data were analyzed using t-test procedure of SAS and the results were presented as mean values± standard error of mean. Significant difference was considered at p<0.05 and a trend toward significance was considered at 0.05<p<0.1. Bioinformatic analysis of the fecal microbiota was carried out using the Majorbio Cloud platform (). Based on the OTU information, alpha diversity indices including Chao1, Shannon, etc. were calculated with Mothur (). The similarity among the microbial communities in different samples was determined by principal coordinate analysis (PCoA) based on Bray-curtis dissimilarity. The linear discriminant analysis (LDA) effect size (LEfSe) () was performed to identify the significantly abundant taxa (phylum to genera) of bacteria among the different groups (LDA score >2, p<0.05).

## RESULTS

### Reproductive performance

The effects of different dietary oils supplementation on reproductive performance in sows were shown in Table 3. There were no differences in reproductive performance such as ADFI, backfat loss, wean-to-estrus interval, born alive, litter birth weight, weaning litter weight, litter weight gain, piglet birth weight, piglet weaning weight and ADG with the two different oils supplementation. However, the number weaned tended to be less (p = 0.06) in the EG than CG.

### Nutrient digestibility

The effects of different dietary oils supplementation on the ATTD of nutrients in sows were shown in Table 4. The ATTD of DM, CP, and GE in sows were increased (p<0.05) with the supplementation of the fatty acid-balanced oil.

### Serum biochemical indices

The effects of different dietary oils supplementation on serum biochemical indexes, liver function, antioxidant indexes, and inflammatory factors in sows and piglets are shown in Table 5 and 6, respectively. The fatty acid-balanced oil increased (p<0.05) serum T-AOC levels of sows on the day of delivery and decreased serum HDL-C and ALB levels of sows on day 21 of lactation (Table 5). Besides, GSH-Px level of sows on day of delivery and IL-8 level of piglets on day 21 of lactation in EG tended to increase (p = 0.07).

### Milk composition

The effects of different dietary oils supplementation on the composition of colostrum and milk in sows are shown in Table 7 and 8, respectively. The fatty acid-balanced oil supplementation decreased (p<0.01) the content of fat in milk and increased (p<0.05) the content of IgG in milk (Table 7). Moreover, the fatty acid-balanced oil supplementation increased (p<0.05) the contents of C15:0, C18:2n6c, C18:3n3, and C22:6n3 in milk and decreased (p<0.05) the contents of C18:1n9c, C20:0, C20:1, C21:0, C20:3n3, C22:1n9 in milk (Table 8). And the contents of C20:4n6 and C22:0 was slightly lower (p<0.10) in EG.

### Fecal microbial composition of piglets

**Figure 1:**
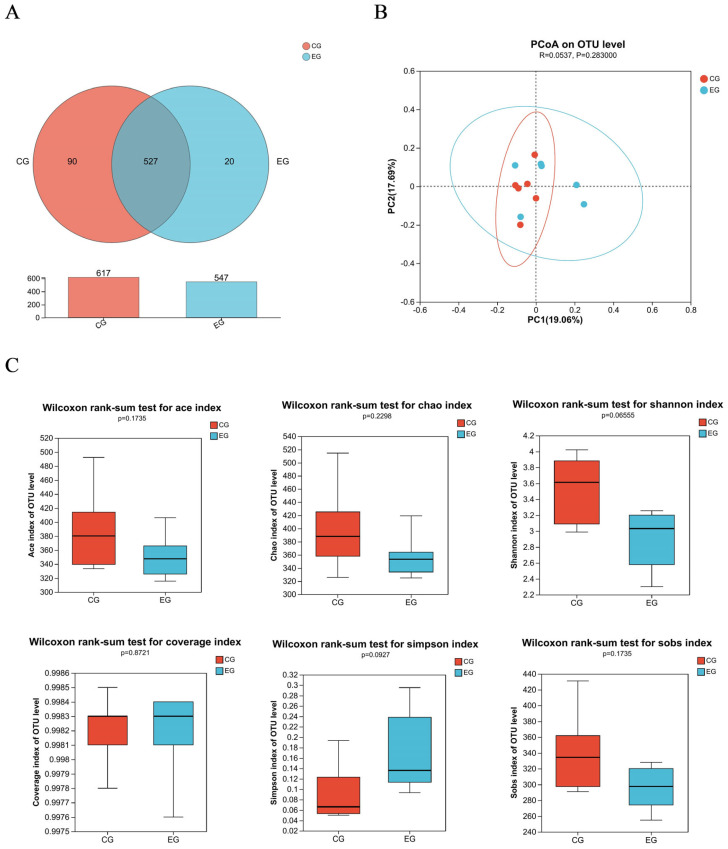
Fecal microbial composition and diversity of weaned piglets among two treatment groups: the control group (CG) fed a basal diet supplemented with 2% soybean oil; and the experiment group (EG) fed the basal diet supplemented with 2% fatty acid-balanced oil. Community composition of fecal bacterial operational taxon of suckling piglets (A), β-diversity analysis by the unweighted version of the UniFrac-based PcoA (B), and Alpha diversity analysis based on Ace, Chao, Shannon, Coverage, Simpson, and Sobs (C).

**Figure 2:**
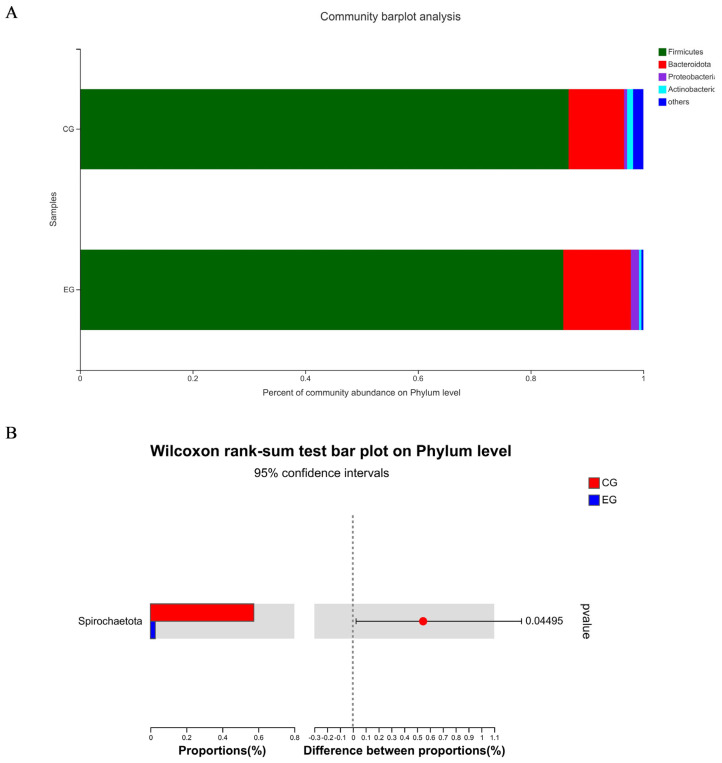
Species composition and differences of fecal microorganisms in weaned piglets at the phylum level among two treatment groups: the control group (CG) fed a basal diet supplemented with 2% soybean oil; and the experiment group (EG) fed the basal diet supplemented with 2% fatty acid-balanced oil. Species composition of fecal microorganisms in weaned piglets at the phylum level (A) and differences of fecal microorganisms in weaned piglets at the phylum level (B).

**Figure 3:**
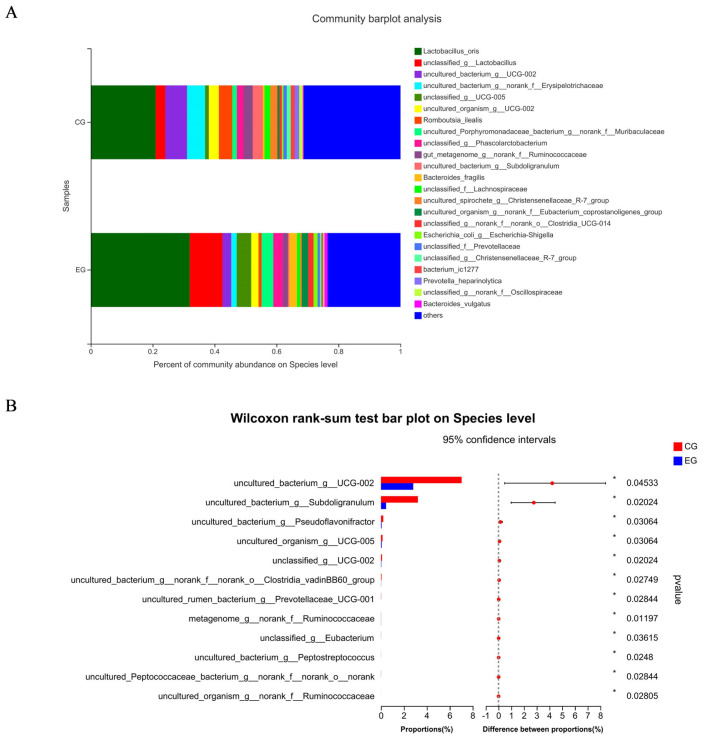
Species composition and differences of fecal microorganisms in weaned piglets at the species level among two treatment groups: the control group (CG) fed a basal diet supplemented with 2% soybean oil; and the experiment group (EG) fed the basal diet supplemented with 2% fatty acid-balanced oil. Species composition of fecal microorganisms in weaned piglets at the species level (A) and differences of fecal microorganisms in weaned piglets at the species level (B).

Figure 1A shows a total of 637 OTUs in the two treatments, 90 unique to CG and 20 unique to EG. The PCoA showed no obvious clustering characteristics in fecal microbial composition between CG and EG (Figure 1B). The lower (p = 0.07) Shannon index and higher (p = 0.09) Simpson index were observed in EG (Figure 1C). Figure 2A shows that *Firmicutes *and *Bacteroidota *were the dominant phyla in the fecal microbial composition of weaned piglets. Figure 2B shows that compared with CG, the average relative abundance of *Spirochaetota* in EG was decreased (p<0.05) by 0.55%. Figure 3A shows that *Lactobacillus_oris* was the most abundant species in the fecal microbial composition of weaned piglets. Figure 3B shows that the average relative abundance of various fecal microorganisms in EG was lower (p<0.05) than that in CG, such as *uncultured_bacterium_g__UCG-002*, *uncultured_organism _g__UCG-005*, and *uncultured_bacterium_g__Subdoligranulum*, etc.

## DISCUSSION

The reproductive performance of sows is a key factor affecting the economic benefits of pig breeding; strengthening nutrition management is an important measure to improve the reproductive performance of sows [16]. Using different fat sources is an important means to improve the reproductive performance of sows and the growth performance of piglets [3]. Different fatty acid composition in diet has been shown in many studies to have an impact on reproductive performance or other important indexes of sows [8–10]. At present, studies on single fats or fatty acids have emerged in an endless stream, such as lauric acid, fish oil, etc. [17,18]. However, studies on balanced fats are more reflected in humans and mice, and rarely reported on pigs. To improve the reproductive performance of lactating sows and the production performance of piglets, this study used a fatty acid-balanced oil instead of soybean oil to investigate the effects of the fatty acid-balanced oil on lactating sows and piglets.

The results showed that the fatty acid-balanced oil had no effect on the reproductive performance of lactating sows, which was consistent with some previous studies [19–21]. The reasons could be too short time of diet treatment before farrowing or no big difference in energy intake. The lower number of piglets weaned was observed in EG, but this may be due to numerically lower born alive but not by treatment effect. However, some studies have shown that different dietary fatty acid compositions had effects on reproductive efficiency, litter size, and wean-to-estrus interval [22–24]. Chen et al [12] reported that the diet of sows supplemented with 1% sodium butyrate or medium-chain fatty acids during late gestation and lactation could improve the feed intake of sows. The fatty acid-balanced oil is supplemented with butyrate and MCFA, but their concentration is well below 1%, which may be the reason why there was no difference in feed intake between CG and EG in this study. In addition, there was no effect of the fatty acid-balanced oil on wean-to-estrus interval in this study, which may be attributed to the overall good body condition recovery and similar energy intake of sows in this study. This result was consistent with the change in the backfat thickness of sows.

The ATTD of energy and nutrients may be affected by many factors, such as chemical composition and processing of feed, animal weight, sex, genotype [25]. Devi et al [26] reported that organic acid and fatty acid mixtures could improve the nutrient digestibility of lactating sows. Li et al [18] reported alpha laurate monoglyceride can increase ATTD of some nutrients. In this study, the fatty acid-balanced oil supplementation improved the ATTD of DM, CP, and GE in sows. The increase in nutrient digestibility might have resulted from the function of butyric acid in promoting intestinal health [12]. The result showed that compared with soybean oil, the fatty acid-balanced oil was beneficial to promote the digestion and absorption of nutrients in lactating sows.

Blood indexes can reflect the health status of pigs. High-density lipoprotein is an anti-atherosclerotic lipoprotein, which is mainly involved in cholesterol metabolism. High-density lipoprotein cholesterol levels are inversely associated with cardiovascular disease risk and can be used to predict risk [27]. Thus, the reduction of HDL may increase cardiovascular disease risk. Albumin is produced by the liver and can transport fatty acids, bile pigments, amino acids, steroid hormones, metal ions, and many therapeutic molecules while maintaining normal osmotic pressure in the blood [28]. However, the fatty acid-balanced oil decreased the serum ALB of sows, indicating decreased liver protein synthesis function. Antioxidant capacity is to protect cells and the body from oxidative stress damage caused by reactive oxygen radicals; T-AOC and GSH-Px can be used to evaluate the antioxidant capacity of bioactive substances [29]. Han et al [30] reported that supplementation with blends of organic acid and MCFAs increased the concentration of serum T-AOC of weaned piglets, indicating that dietary fatty acid composition affected the antioxidant capacity of the body. In this study, the supplementation of the fatty acid-balanced oil increased T-AOC levels, and GSH-Px tended to increase, indicating that the fatty acid-balanced oil can improve the antioxidant capacity of lactating sows. Interleukin-8, a pro-inflammatory cytokine, serves as an important role in mediating inflammatory and immune responses [31]. The trend of IL-8 suggested that dietary fatty acid composition in sows might influence the inflammatory response of offspring, but no adverse effects on piglets were observed during the experiment.

Milk is the main nutrient source of suckling piglets, and immunoglobulins in milk play an active role in the establishment of the passive immune system of piglets [32]. In this study, it was found that IgG content in milk was increased, and milk fat content was decreased in sows with the fatty acid-balanced oil supplementation. Jin et al [4] reported fish oil rich in eicosapentaenoic acid (EPA) and docosahexenoic acid (DHA) could increase the IgG content in milk. Besides, Shen et al [33] reported that the supplementation with olive oil to sow diets reduced the fat content in colostrum and milk of sows. Chen et al [12] reported butyrate supplementation increased the IgG content in milk. These results suggest that dietary fats and fatty acids can affect milk composition. In this study, the fatty acid-balanced oil improved the content of immunoglobulin in sow’s milk, but reduced the content of fat in milk, and its mechanism needs to be further studied.

In this study, the fatty acid composition of milk in lactating sows was affected by the supplementation of the fatty acid-balanced oil. Shen et al [33] and Lauridsen et al [34] reported that fatty acid composition in colostrum and milk could be affected by the supplementation of fat during late gestation and lactation. Zhe et al [3] reported the fatty acid content in diet was positively correlated with the fatty acid yield in milk. Thus, different dietary fatty acid composition may result in altered fatty acid composition in milk. However, there are many fatty acids in milk that did not reflect the composition of fatty acid-balanced oil (C18:2, C18:3, C20:1, C21:0, C20:4, C20:3, and C22:1), probably because of too little variation in fatty acids between the two diets [3]. In addition, fatty acid composition of milk may be affected by the different blood indices of the two treatments [35].

Healthy growth of piglets requires a dynamic and balanced intestinal micro-ecological environment, because healthy intestinal flora can promote the nutritional metabolism of pigs, maintain the intestinal mucosal barrier, regulate immune response, inhibit pathogen infection, and other functions [36]. In this study, trends in alpha diversity indices were observed, suggesting fatty acid-balanced oil supplementation could affect fecal microorganism of piglets. *Firmicutes* and *Bacteroidota *are the dominant phyla in the fecal microbial composition of suckling piglets, accounting for more than 90% of the intestinal flora of piglets, consistent with this study; the two phyla are beneficial for piglets to create and maintain an anaerobic environment in the intestine [37]. The high relative abundance of *Spirochaetota *will lead to intestinal microbiota imbalance and intestinal inflammation [38]. In this study, the fatty acid-balanced oil decreased the abundance of *Spirochaetota* in the feces of suckling piglets, which may be beneficial for the piglets to maintain the balance of intestinal microbiota. This result may be attributed to the butyric acid in the fatty acid-balanced oil [39]. Consistent with the result of this study, one of the most abundant microbiotas of the pig is the lactic acid bacteria, in particular *lactobacilli*, which could improve intestinal physiology, regulate the intestinal immune system, and maintain the intestinal ecology of the host [40]. The higher relative abundances of *UCG-002* and *UCG-005* in EG will result in a higher concentration of lithocholic acid, which is a toxic bile acid [41] and *Subdoligranulum *is related to rheumatoid arthritis [42]. Therefore, the fatty acid-balanced oil decreased the relative abundance of some potentially pathogenic microorganisms. However, whether the reduction of multiple microorganisms is beneficial to piglets remains to be further studied. It was indicated that the fatty acid-balanced oil changed the intestinal microbial structure and diverse composition of suckling piglets to some extent.

In conclusion, the present study indicates that the fatty acid-balanced oil improved nutrient digestibility, and changed milk composition, which may contribute to the fecal microbial composition of piglets. Further study is needed to investigate more ideal fatty acid composition of oil in lactating sows.

## Figures and Tables

**Table 1:** The composition of the soybean oil and the fatty acid-balanced oil (mg/g)

Items	Formulas	Soybean oil	Fatty acid-balanced oil
Butyric acid	C4:0	-	77.3
Lauric acid	C12:0	-	0.10
Tridecanoic acid	C13:0	-	0.04
Myristic acid	C14:0	0.73	3.60
Pentadecylic acid	C15:0	0.14	0.50
Palmitic acid	C16:0	102.90	94.51
Palmitoleic acid	C16:1	0.75	3.48
Margaric acid	C17:0	0.94	1.17
Stearic acid	C18:0	39.93	34.22
Oleic acid	C18:1n9c	194.03	167.61
Linoleic acid	C18:2n6c	517.03	440.02
α-Linolenic acid	C18:3n3	74.32	53.85
Arachidic acid	C20:0	3.37	3.12
Eicosenoic acid	C20:1	1.75	2.58
Heneicosanoic acid	C21:0	0.41	0.46
11C, 14C-Eicosadienoic acid	C20:2	0.36	0.27
g-Eicosatrienoic acid	C20:3n6	-	0.07
Arachidonic acid	C20:4n6	-	0.52
Eicosatrienoic	C20:3n3	-	0.07
Behenic acid	C22:0	3.38	2.74
Eicosapentaenoic acid	C20:5n3	-	4.33
Erucic acid	C22:1n9	0.11	0.21
Tricosanoic acid	C23:0	0.55	0.53
Lignoceric acid	C24:0	1.35	1.63
Nervonic acid	C24:1	-	0.36
Docosahexaenoic acid	C22:6n3	-	6.78
SFA	-	153.70	219.93
MUFA	-	196.65	174.23
PUFA	-	591.70	505.90
n-6 PUFA	-	517.03	440.61
n-3 PUFA	-	74.32	65.03
n-6:n-3	-	6.96	6.78

SFA, saturated fatty acid; MUFA, monounsaturated fatty acid; PUFA, polyunsaturated fatty acid.

**Table 2:** Ingredient and chemical composition of the experimental diets^1)^ (as-fed basis)

Items	CG	EG
Ingredients (%)		
Corn	63.92	63.92
Soybean meal (CP 43%)	28.00	28.00
Wheat bran	3.00	3.00
Soybean oil	2.00	-
Fatty acid-balanced oil	-	2.00
Dicalcium phosphate	1.38	1.38
Limestone	0.90	0.90
Salt	0.30	0.30
Premix^2)^	0.50	0.50
Nutrient levels^3)^		
Digestible energy (MJ/kg)	15.51	15.51
Crude protein (%)	17.90	17.90
Calcium (%)	0.79	0.79
Available phosphorus (%)	0.39	0.39
SID Lysine (%)	0.86	0.86
SID Methionine (%)	0.27	0.27
SID Threonine (%)	0.58	0.58
SID Tryptophan (%)	0.16	0.16

CP, crude protein; SID, standardized ileal digestible.

1) CG, sows in the control group fed a basal diet supplemented with 2% soybean oil; EG, sows in the experiment group fed the basal diet supplemented with 2% fatty acid-balanced oil.

2) The premix provided the following per kilogram of diet: vitamin A, 12,000 IU; vitamin E, 24 IU; vitamin K_3_, 2.0 mg; vitamin B_6_, 3.0 mg; vitamin B_12_, 24 μg; vitamin B_2_, 6.0 mg; vitamin B_1_, 2.0 mg; nicotinic acid, 30 mg; D-pantothenic acid, 20 mg; folic acid, 3.6 mg; biotin, 0.1 mg; choline chloride, 0.4 mg; Cu, 8.0 mg as CuSO_4_·5H_2_O; Fe, 96 mg as FeSO_4_·H_2_O; Zn, 120 mg as ZnSO_4_·H_2_O; Mn, 40 mg as MnSO_4_·H_2_O; I, 0.56 mg as Ca(IO_3_)_2_; Se, 0.4 mg as sodium selenite.

3) Nutrient levels are calculated according to the NRC (2012).

**Table 3:** Effects of different dietary oils supplementation on reproductive performance in sows^1)^

Items	CG	EG	SEM^2)^	p-value
Sows				
Parity	4.92	5.00	0.56	0.942
ADFI (kg/d)	5.59	5.56	0.09	0.858
Backfat loss (mm)	1.72	1.20	0.24	0.274
Wean-to-estrus interval (d)	4.17	4.29	0.12	0.646
Piglets				
Born alive (n)	13.8	13.0	0.60	0.510
Number weaned (n)	12.0	11.3	0.19	0.056
Litter birth weight (kg)	19.7	19.6	0.79	0.933
Weaning litter weight (kg)	66.3	66.2	2.39	0.979
Litter weight gain (kg)	46.6	46.6	2.26	0.968
Piglet birth weight (kg)	1.5	1.5	0.04	0.474
Piglet weaning weight (kg)	5.5	5.9	0.20	0.314
ADG (g/d)	193	209	8.67	0.360

ADFI, average daily feed intake; ADG, average daily gain.

1) CG, sows in the control group fed a basal diet supplemented with 2% soybean oil; EG, sows in the experiment group fed the basal diet supplemented with 2% fatty acid-balanced oil.

2) SEM, standard error of the mean (n = 12).

**Table 4:** Effects of different dietary oils supplementation on ATTD of nutrients in sows^1)^

Items	CG	EG	SEM^2)^	p-value
DM (%)	88.7	90.3	0.44	0.026
CP (%)	89.4	90.7	0.36	0.024
CF (%)	50.5	57.4	2.77	0.109
GE (%)	88.2	90.2	0.52	0.031

ATTD, apparent total tract digestibility; DM, dry matter; CP, crude protein; CF, crude fat; GE, gross energy.

1) CG, sows in the control group fed a basal diet supplemented with 2% soybean oil; EG, sows in the experiment group fed the basal diet supplemented with 2% fatty acid-balanced oil.

2) SEM, standard error of the mean (n = 6).

**Table 5:** Effects of different dietary oils supplementation on serum biochemical indices, liver function, antioxidant indices, and inflammatory factors in sows^1)^

Items	The day of delivery				Day 21 of lactation			
	
	CG	EG	SEM^2)^	p-value	CG	EG	SEM	p-value
Serum biochemical indices								
TG (mmol/L)	0.16	0.15	0.03	0.892	0.09	0.16	0.02	0.051
TC (mmol/L)	0.93	1.01	0.11	0.652	1.70	1.53	0.08	0.161
HDL-C (mmol/L)	0.39	0.45	0.29	0.174	0.89	0.74	0.04	0.044
LDL-C (mmol/L)	0.62	0.65	0.62	0.769	0.71	0.73	0.04	0.669
ALB (g/L)	39.41	38.57	0.88	0.510	41.80	39.38	0.69	0.033
TP (g/L)	71.37	64.88	3.04	0.162	76.52	75.58	2.32	0.782
Liver functions								
AST (U/L)	39.50	41.50	3.95	0.728	22.83	24.67	2.15	0.559
ALT (U/L)	35.50	42.00	3.24	0.187	34.17	38.00	4.43	0.554
Antioxidant indices								
GSH-Px (U/mL)	916.08	936.16	7.07	0.073	848.03	860.99	17.20	0.606
T-AOC (U/mL)	12.51	12.89	0.12	0.049	11.45	11.64	0.26	0.625
SOD (U/mL)	128.35	132.22	1.63	0.123	114.53	117.19	3.42	0.595
CAT (U/mL)	9.89	10.15	0.11	0.126	8.93	9.14	0.23	0.527
MDA (nmol/mL)	4.87	4.68	0.09	0.144	5.51	5.39	0.17	0.628
IgA (g/L)	1.29	1.34	0.02	0.128	1.12	1.15	0.05	0.605
IgG (g/L)	8.96	9.00	0.05	0.497	8.67	8.71	0.05	0.662
IgM (g/L)	0.96	0.98	0.01	0.214	0.87	0.88	0.02	0.689
Inflammatory factors								
TNF-α (pg/mL)	23.46	18.66	8.97	0.713	12.91	9.36	5.31	0.646
IL-8 (pg/mL)	10.31	5.16	3.62	0.338	3.81	2.50	0.81	0.277
IL-6 (pg/mL)	18.39	35.38	18.25	0.525	11.10	13.95	8.16	0.810
IL-10 (pg/mL)	31.34	6.22	12.15	0.175	4.02	3.42	2.03	0.840

TG, triglyceride; TC, total cholesterol; HDL-C, high-density lipoprotein cholesterol; LDL-C, low-density lipoprotein cholesterol; ALB, albumin; TP, total protein; AST, aspartate aminotransferase; ALT, alanine aminotransferase; GSH-Px, glutathione peroxidase; T-AOC, total antioxidant capacity; SOD, superoxide dismutase; CAT, catalase; MDA, malondialdehyde; IgA, immunoglobulin A; TNF-α, tumor necrosis factor-α; IL-8, interleukin-8.

1) CG, sows in the control group fed a basal diet supplemented with 2% soybean oil; EG, sows in the experiment group fed the basal diet supplemented with 2% fatty acid-balanced oil.

2) SEM, standard error of the mean (n = 6).

**Table 6:** Effects of different dietary oils supplementation on serum biochemical indices, liver functions, antioxidant indices, and inflammatory factors in piglets^1)^

Items	CG	EG	SEM^2)^	p-value
Serum biochemical indices				
TG (mmol/L)	0.95	1.06	0.19	0.686
TC (mmol/L)	3.84	4.31	0.43	0.462
HDL-C (mmol/L)	1.63	1.77	0.11	0.377
LDL-C (mmol/L)	1.87	2.03	0.28	0.697
ALB (g/L)	34.60	32.42	1.11	0.195
TP (g/L)	51.30	49.92	1.23	0.444
Liver functions				
AST (U/L)	38.33	36.50	2.71	0.643
ALT (U/L)	33.00	28.83	2.85	0.325
Antioxidant indices				
GSH-Px (U/mL)	817.15	795.12	13.60	0.279
T-AOC (U/mL)	11.02	10.59	0.238	0.223
SOD (U/mL)	108.81	103.78	2.88	0.245
CAT (U/mL)	8.62	8.24	0.19	0.194
MDA (nmol/mL)	5.78	6.05	0.14	0.208
IgA (g/L)	1.04	0.98	0.04	0.238
IgG (g/L)	8.61	8.52	0.05	0.207
IgM (g/L)	0.83	0.80	0.02	0.238
Inflammatory factors				
TNF-α (pg/mL)	7.40	5.10	2.18	0.472
IL-8 (pg/mL)	1.35	2.07	0.24	0.065
IL-6 (pg/mL)	3.04	2.15	0.63	0.347
IL-10 (pg/mL)	1.41	0.97	0.73	0.678

TG, triglyceride; TC, total cholesterol; HDL-C, high-density lipoprotein cholesterol; LDL-C, low-density lipoprotein cholesterol; ALB, albumin; TP, total protein; AST, aspartate aminotransferase; ALT, alanine aminotransferase; GSH-Px, glutathione peroxidase; T-AOC, total antioxidant capacity; SOD, superoxide dismutase; CAT, catalase; MDA, malondialdehyde; IgA, immunoglobulin A; TNF-α, tumor necrosis factor-α; IL-8, interleukin-8.

1) CG, sows in the control group fed a basal diet supplemented with 2% soybean oil; EG, sows in the experiment group fed the basal diet supplemented with 2% fatty acid-balanced oil.

2) SEM, standard error of the mean (n = 6).

**Table 7:** Effects of different dietary oils supplementation on the composition of colostrum and milk in sows^1)^

Items	CG	EG	SEM^2)^	p-value
Colostrum				
Milk fat (%)	4.83	4.93	0.23	0.776
Lactoprotein (%)	14.60	12.53	1.12	0.218
Lactose (%)	2.91	3.39	0.26	0.218
IgA (g/L)	5.48	4.47	0.55	0.224
IgG (g/L)	34.93	30.57	2.34	0.218
IgM (g/L)	6.68	6.14	0.47	0.235
Milk				
Milk fat (%)	5.13	4.28	0.17	0.006
Lactoprotein (%)	5.46	7.55	1.24	0.260
Lactose (%)	6.28	5.63	0.42	0.297
IgA (g/L)	2.70	2.83	0.12	0.463
IgG (g/L)	22.25	24.16	0.50	0.022
IgM (g/L)	4.65	4.79	0.06	0.112

IgA, immunoglobulin A.

1) CG, sows in the control group fed a basal diet supplemented with 2% soybean oil; EG, sows in the experiment group fed the basal diet supplemented with 2% fatty acid-balanced oil.

2) SEM, standard error of the mean (n = 6).

**Table 8:** Effects of different dietary oils supplementation on fatty acid composition in colostrum and milk in sows^1)^

Items	Colostrum				Milk			
	
	CG	EG	SEM^2)^	p-value	CG	EG	SEM^2)^	p-value
C6:0	0.00	0.00	0.00	-	0.03	0.03	0.00	0.600
C8:0	0.00	0.00	0.00	-	0.03	0.03	0.00	1.000
C10:0	0.00	0.00	0.00	-	0.18	0.18	0.01	0.938
C12:0	0.19	0.22	0.03	0.490	0.29	0.31	0.02	0.615
C14:0	2.04	0.14	0.09	0.438	3.71	3.84	0.17	0.582
C14:1	0.03	0.03	0.00	0.600	0.27	0.31	0.02	0.221
C15:0	0.19	0.20	0.02	0.663	0.08	0.10	0.01	0.021
C16:0	23.84	25.21	0.64	0.161	33.27	33.82	0.59	0.518
C16:1	3.18	3.10	0.28	0.853	9.75	10.48	0.37	0.191
C17:0	0.36	0.41	0.02	0.212	0.14	0.16	0.01	0.171
C18:0	5.47	6.33	0.34	0.102	4.32	3.96	0.15	0.122
C18:1n9c	31.65	29.88	1.49	0.420	27.32	23.82	0.95	0.026
C18:2n6c	26.62	26.50	1.10	0.943	16.89	19.21	0.56	0.016
C18:3n3	2.04	1.87	0.09	0.224	1.45	1.75	0.08	0.020
C20:0	0.15	0.17	0.01	0.021	0.13	0.12	0.00	0.028
C20:1	0.34	0.30	0.03	0.412	0.31	0.21	0.02	0.017
C21:0	0.67	0.65	0.02	0.474	0.39	0.31	0.02	0.049
C20:3n6	0.35	0.34	0.03	0.815	0.11	0.10	0.01	0.511
C20:4n6	1.12	0.97	0.07	0.170	0.49	0.43	0.02	0.094
C20:3n3	0.15	0.15	0.01	0.718	0.09	0.07	0.01	0.040
C22:0	0.21	0.20	0.02	0.726	0.21	0.16	0.02	0.096
C20:5n3	0.24	0.22	0.02	0.341	0.06	0.07	0.00	0.260
C22:1n9	0.07	0.06	0.00	0.323	0.07	0.05	0.00	0.014
C22:2	0.08	0.09	0.01	0.646	0.07	0.06	0.01	0.625
C23:0	0.05	0.05	0.00	0.134	0.04	0.05	0.00	0.141
C24:0	0.28	0.33	0.03	0.314	0.13	0.14	0.01	0.375
C24:1	0.20	0.17	0.01	0.075	0.12	0.12	0.01	0.886
C22:6n3	0.49	0.44	0.05	0.427	0.06	0.11	0.00	<0.001

1) CG, sows in the control group fed a basal diet supplemented with 2% soybean oil; EG, sows in the experiment group fed the basal diet supplemented with 2% fatty acid-balanced oil. The unit (mg/g) is for fatty acid composition.

2) SEM, standard error of the mean (n = 6).
